# Authors’ reply

**Published:** 2010

**Authors:** Rakesh K. Chawla, Arun Madan, Dinesh Mehta, Kiran Chawla

**Affiliations:** *Department of Respiratory Medicine, Critical Care and Sleep Disorders, Jaipur Golden Hospital, New Delhi, India. E-mail: chawla.rakesh@rediffmail.com*

Sir,

Answer to the questions[1] posed on the paper “Controlling hemoptysis: An alternative approach” published in April 2010 issue of Lung India.[2]

We would like to thank the reader[1] for showing interest in our article.[2] Our response to queries is as follows:

Query no 1:

We want to make it clear that we did not use BAE prophylactically in this patient. The message taken by the reader may not be right because we have used BAE as a therapeutic procedure to control hemoptysis that helped him in two ways-

First, we achieved the therapeutic effect by controlling his hemoptysis. Second, we were able to diagnose the case also after doing bronchial biopsy. So it was not prophylactic BAE in the true sense but an alternative to control hemoptysis when conservative methods failed. Nowhere in our article had we claimed that this was a novel approach. We hope that we have clarified question number b) ii and c which also address the same issue.

Query no 2:

We have discussed the issue raised by the reader with the radiologist. According to him, he had given the report only after reconfirming by using the software to reconstruct the images of angiogram with same contrast CT.

Query no 3:

In the lung, there are two main indications for embolization in relation to hemorrhage: (1) pulmonary arteriovenous malformations (AVMs) and (2) hemoptysis.[3] Our aim of describing the pros and cons of the procedure in ‘Discussion’ was to improve awareness of readers about evidence-based literature regarding the process which could be clinically useful.

Thanks,
